# Circadian clock disruption by selective removal of endogenous carbon monoxide

**DOI:** 10.1038/s41598-018-30425-6

**Published:** 2018-08-10

**Authors:** Saika Minegishi, Ikuko Sagami, Shigeru Negi, Koji Kano, Hiroaki Kitagishi

**Affiliations:** 1 Department of Molecular Chemistry and Biochemistry, Faculty of Science and Engineering, Doshisha University, Kyotanabe, Kyoto 610-0321 Japan; 2 Graduate School of Life and Environmental Sciences, Kyoto Prefectural University, Sakyo-ku, Kyoto 606-8522 Japan; 3 Faculty of Pharmaceutical Sciences, Doshisha Women’s College of Liberal Arts, Kyotanabe, Kyoto 610-0395 Japan

## Abstract

Circadian rhythms are regulated by transcription-translation feedback loops (TTFL) of clock genes. Previous studies have demonstrated that core transcriptional factors, NPAS2 and CLOCK, in the TTFL can reversibly bind carbon monoxide (CO) *in vitro*. However, little is known about whether endogenous CO, which is continuously produced during a heme metabolic process, is involved in the circadian system. Here we show that selective removal of endogenous CO in mice considerably disrupts rhythmic expression of the clock genes. A highly selective CO scavenger, hemoCD1, which is a supramolecular complex of an iron(II)porphyrin with a per-*O*-methyl-β-cyclodextrin dimer, was used to remove endogenous CO in mice. Intraperitoneal administration of hemoCD1 to mice immediately reduced the amount of internal CO. The removal of CO promoted the bindings of NPAS2 and CLOCK to DNA (E-box) in the murine liver, resulting in up-regulation of the E-box-controlled clock genes (*Per1*, *Per2*, *Cry1*, *Cry2*, and *Rev-erbα*). Within 3 h after the administration, most hemoCD1 in mice was excreted in the urine, and heme oxygenase-1 (HO-1) was gradually induced in the liver. Increased endogenous CO production due to the overexpression of HO-1 caused dissociation of NPAS2 and CLOCK from E-box, which in turn induced down-regulation of the clock genes. The down-regulation continued over 12 h even after the internal CO level recovered to normal. The late down-regulation was ascribed to an inflammatory response caused by the endogenous CO reduction. The CO pseudo-knockdown experiments provided the clear evidence that endogenous CO contributes to regulation in the mammalian circadian clock.

## Introduction

**Figure 1:**
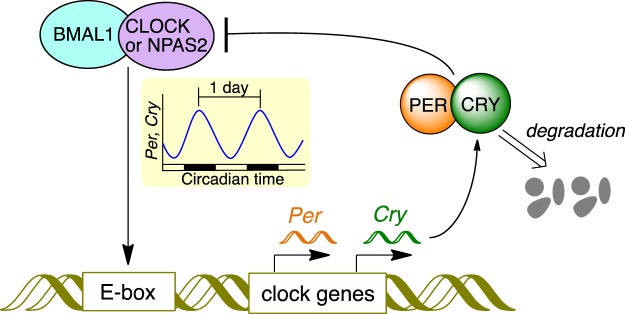
The core transcription-translation feedback loop in the circadian system of mammalian cells. The components of Fig. 1 were drawn using ChemBioDraw.

The circadian rhythm is a naturally occurring day-and-night oscillation system that controls physiological and behavioral cycles and is regulated in almost all cells^1–4^. Figure 1 depicts the core circadian clock system in mammalian cells. This system involves a transcription-translation feedback loop (TTFL) for the rhythmic expression of clock components on approximately 24 h cycles^1–4^. CLOCK and NPAS2 are the transcriptional factors that play central and overlapping roles in the TTFL^1–7^. Both proteins form heterodimers with BMAL1 and bind to a specific DNA sequence called E-box. Transcription of clock genes, such as *periods* (*Per*) and *cryptochromes* (*Cry*), is enhanced by these heterodimers. The translated PER and CRY proteins also form a heterodimer, which acts on BMAL1:CLOCK(NPAS2) to repress the transcription of *Per* and *Cry*. Both PER and CRY proteins are gradually degraded through the post-transcriptional modifications such as phosphorylation and ubiquitination^8^, and then transcription of *Per* and *Cry* restarts, thus completing the TTFL.

Carbon monoxide (CO) is continuously produced in mammalian cells. The major source of endogenous CO is the degradation of heme by heme oxygenases (HOs)^9,10^. Similar to nitric oxide (NO) and hydrogen sulfide (H_2_S), CO acts as a gaseous signal messenger with several protective effects, such as anti-inflammation, anti-apoptosis, and anti-proliferation^9–12^. In parallel with physiological studies on CO, CO-responsive transcriptional factors have been identified, such as NPAS2^13^, CooA^14^, and RcoM^15,16^. In contrast to NO and H_2_S, which are highly reactive to metal ions, thiol groups in cysteine residues, and molecular oxygen, CO is relatively inert and only reactive to metal cofactors, mostly ferrous heme, in biological systems^16–18^; therefore, these CO-sensing proteins possess heme cofactors, and binding of CO to the heme induces structural/functional changes of the proteins. Dioum *et al*. demonstrated that the BMAL1:NPAS2 heterodimer loses its DNA-binding character *in vitro* in the presence of CO^13^. The CLOCK protein also possesses heme as a prosthetic cofactor and the heme-based sensing function toward CO is suggested^19^. Therefore, endogenous CO might participate in the regulation of the TTFL in the circadian clock system^20^. Tu and McKnight early demonstrated that endogenous/exogenous CO plays a regulatory role in the yeast metabolic cycle^21^. A recent pathological study showed that application of exogenous CO could adjust the disrupted circadian rhythms in injured cells^22,23^. The role of endogenous CO in the circadian clock system has been recently studied by using HO-knockout/knockdown systems^24^, suggesting that rhythmic heme degradation generating endogenous CO is required for keeping the E-box-controlled circadian rhythms. However, heme itself, whose internal level is controlled by the HO activity, has an effect on the regulation of circadian rhythms^25,26^. In addition, heme degradation by HO consumes NADPH and generates not only CO, but also biliverdin and iron^9,10^. Intracellular NADPH level is also an influencing factor in the regulation of circadian rhythms^27,28^. Genetic or pharmacological inhibition of the HO activity has the potential to affect various biological events in addition to CO-depletion. Thus, the use of a selective CO-removal agent would be more favorable to clarify the contribution of endogenous CO to the circadian clock.

**Figure 2:**
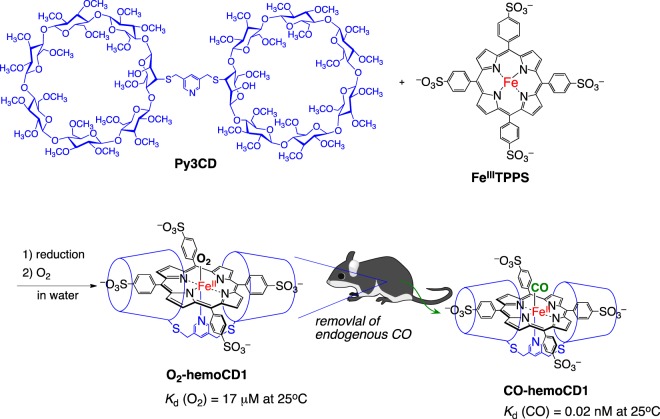
The structure of a CO-scavenging agent, hemoCD1, a 1:1 supramolecular inclusion complex of an ironporphyrin (FeTPPS) and a per-*O*-methylated cyclodextrin dimer (Py3CD). HemoCD1 is used for selective removal of endogenous CO in mice *via* the ligand exchange reaction of O_2_ with CO. The binding affinity for CO (*P*_1/2_^CO^ = 0.000015 Torr) is much higher than that for O_2_ (*P*_1/2_^O2^ = 10 Torr)^34^. The components of Fig. 2 were drawn using ChemBioDraw.

In this report, we show that selective removal of endogenous CO in mice significantly affects the expression levels of the E-box-controlled clock genes in the murine liver. We utilized hemoCD1, a highly selective CO scavenging agent working in aqueous media. HemoCD1 (Fig. 2) is a very stable 1:1 supramolecular inclusion complex comprised of 5,10,15,20-tetrakis(4-sulfonatophenyl)porphinatoiron(II) (Fe^II^TPPS) encapsulated by a per-*O*-methylated β-cyclodextrin dimer with a pyridine ligand (Py3CD). Our group has extensively studied hemoCD1 as a water-soluble hemoprotein model compound^29–32^. Similar to native hemoglobin and myoglobin, hemoCD1 reversibly binds oxygen (O_2_) and CO in aqueous solutions at ambient temperature. It is noteworthy that the CO binding affinity of hemoCD1 is extremely high (*K*_d_ = 0.02 nM at 25 °C), approximately 100 times higher than that of hemoglobin in the R-state in aqueous solutions, whereas the O_2_ binding affinity of hemoCD1 is moderate and close to that of hemoglobin in the T-state^30,33,34^. To the best of our knowledge, the CO-binding affinity of hemoCD1 in aqueous solutions is the highest among the reported CO-binding hemoproteins. More advantageously, the NO-binding affinity of hemoCD1 is lower than that of hemoglobin^35^. The coordination strength of H_2_S to a hemoCD1 analog is also relatively weak, and the SH^–^ ligand is easily replaceable with CO^36^. Therefore, it might be possible to use hemoCD1 to selectively remove internal CO in mammals, creating an endogenous CO pseudo-knockdown state *in vivo*^33–35^. The CO binding affinity of hemoCD1 is also much higher than those of NPAS2 (*K*_d_ = 1–2 μM at 25 °C)^13^ and CLOCK (PAS-A domain, *K*_d_ = ca. 0.1 mM at 22 °C)^19^. Using hemoCD1, therefore, it become possible to study of the effects of endogenous CO on the circadian clock system. Here, the pseudo-knockdown study for CO *in vivo* provides the clear experimental evidence that endogenous CO contributes to regulation of the mammalian circadian clock through acting on NPAS2 and CLOCK and modulating the clock genes related to inflammatory responses.

## Results

### Administration of hemoCD1 in mice

Before doing experiments, the mice were housed for two weeks under 12 h light/dark (LD) cycle (lights on at 7:00 and off at 19:00) with free feeding. From the day before hemoCD1 administration to the end of observation, the mice were housed under constant dark (DD) conditions without feeding to prevent the entrainment of their internal clocks by external stimuli. A solution containing hemoCD1 (1.0 mM), which existed as the O_2_ adduct (O_2_-hemoCD1) under aerobic conditions, in phosphate-buffered saline (PBS, 0.15 mL) was intraperitoneally (i.p.) administered to the mice. After the i.p. administration of hemoCD1, endogenous CO in the mice was bound to hemoCD1 *via* ligand exchange with O_2_ and excreted in the urine in a form complexed with hemoCD1 (CO-hemoCD1) as previously demonstrated^34^. The amount of hemoCD1 administered was sufficient to deplete the endogenous CO in mice; the excreted amount of endogenous CO as CO-hemoCD1 was saturated when hemoCD1 was administered at concentrations higher than 1.0 mM (0.15 mL)^34^. Throughout this study, we used mice treated with buffer (PBS) and the free-base complex of hemoCD1 (hemoCD1 without Fe^II^, Fb-hemoCD1) as negative controls. In addition, we tested CO-hemoCD1 in place of O_2_-hemoCD1. Such a CO-adduct never altered the concentration of CO in mice and showed no effect on clock gene expression (Fig. S1). A series of control experiments confirmed that the changes in clock gene expression observed in the hemoCD1-treated mice must have arisen from the lack of endogenous CO.

### Effects of endogenous CO depletion on the clock gene expression

**Figure 3:**
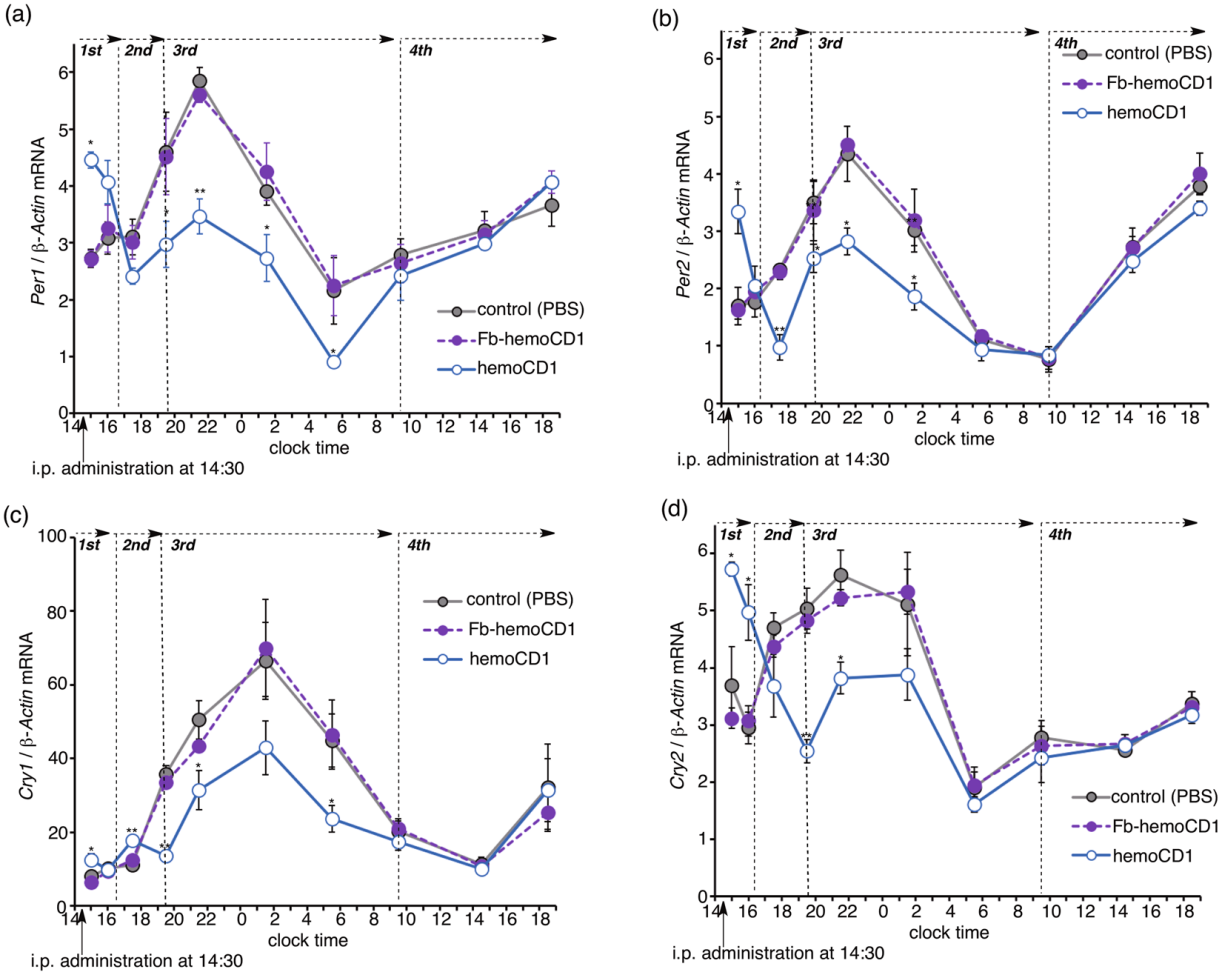
Changes in the mRNA levels of clock genes (*Per1*, **a**; *Per2*, **b**; *Cry1*, **c**; *Cry2*, **d**) in the murine liver after i.p. administration of hemoCD1 at clock time 14.5 (14:30). PBS and Fb-hemoCD1 were similarly administered as control samples. Each bar represents the mean ± SE (*n* = 3 mice per group). The mice were housed for two weeks under a 12 h light/dark (LD) cycle (lights on at 7:00 and light off at 19:00) until the day before the experiments. The mice were then housed under constant dark (DD) conditions during the observations. Asterisk denotes statistical significance (^*^P < 0.05, ^**^P < 0.01), as compared to the controls. Note that the value on the vertical axis cannot be directly compared between the different panels (a–d) because the amount of cDNA used for real-time PCR varies with the gene of interest.

After i.p. administration of hemoCD1 to mice at 14:30, the expression levels of the clock genes *Per1*, *Per2*, *Cry1*, and *Cry2* were measured by quantitative real-time PCR (RT-PCR) at different time points. The time profiles for the mRNA of the clock genes in the livers of the hemoCD1-treated mice were obviously different from those in the livers of the control groups (Fig. 3). The mRNA levels initially increased at clock time 15 (0.5 h after the administration), and then started to decline to levels lower than those in the control groups. The down-regulation of the clock genes continued until around 9:30 on day two (19 h after the administration). In addition, no significant changes in the mRNA levels were observed for the brains of the hemoCD1-treated mice (Fig. S2). HemoCD1 does not remove endogenous CO in the brain (Fig. S3), probably due to poor slipping ability of hemoCD1 through the blood brain barrier. This result suggests that administration of hemoCD1 to mice cannot possibly affect the master clock of the suprachiasmatic nucleus.

**Figure 4:**
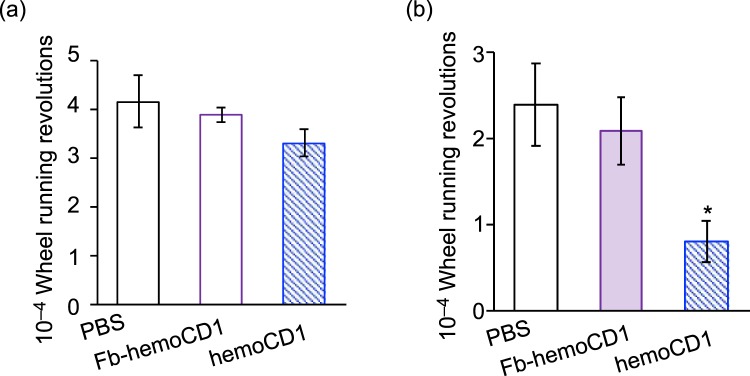
Cumulative wheel running revolutions in the first (**a**) and second 24 h periods (**b**) after i.p. administration of PBS, Fb-hemoCD1, and hemoCD1. Each bar represents the mean ± SE (*n* = 3 mice per group). The mice were housed for two weeks under a 12 h light/dark (LD) cycle (lights on at 7:00 and light off at 19:00) until the day before the experiments. The mice were then housed under constant dark (DD) conditions during the observations. Asterisk denotes statistical significance (^*^P < 0.05), as compared to the controls. The raw actograms for measuring the wheel running activity are shown in Fig. S4.

The disruption of clock gene expression affected the physical behavior of the mice. Analysis of wheel-running activity of mice (Fig. 4) showed that administration of hemoCD1 resulted in impaired locomotor action. This effect reached statistical significance on the second day after the administration. This observed behavioral effect is likely related to the down-regulation of *Cry* expression, as previously reported for *Cry*-null mice^37^ and/or to the CO-removal-induced inflammatory response that increases TNF-α levels in the livers of mice during the third phase (*vide infra*).

### CO depletion-induced activity changes in NPAS2 and CLOCK

**Figure 5:**
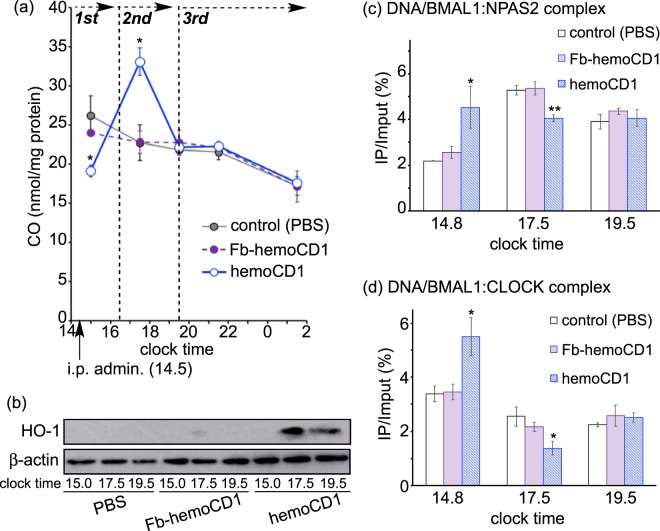
Quantification of CO, HO-1, and the DNA/protein complexes in the liver of the hemoCD1-treated mice. The mice were housed for two weeks under a 12 h light/dark (LD) cycle (lights on at 7:00 and light off at 19:00) until the day before the experiments. The mice were then housed under constant dark (DD) conditions during the observations. (**a**) Endogenous CO levels were measured by the assay using hemoCD1^35^. (**b**) HO-1 levels were ascertained by western blotting with an anti-HO-1 antibody at clock time 15.0 (first phase), 17.5 (second phase), and 19.5 (third phase). (**c**,**d**) ChIP assays were conducted using anti-NPAS2 and anti-CLOCK antibodies to measure the corresponding DNA complexes at clock time 14.8 (first phase), 17.5 (second phase), and 19.5 (third phase). PBS and Fb-hemoCD1 were used as controls. Each bar represents the mean ± SE (*n* = 3 mice per group). Asterisk denotes statistical significance (^*^P < 0.05, ^**^P < 0.01), as compared to the controls.

To clarify the mechanism for the circadian rhythm disruption observed in the CO-reduced mice, we divided the mRNA expression profiles into four phases, as shown in Fig. 3. In the first phase (clock time 14.5–16.5), i.p. administration of hemoCD1 derived higher clock gene mRNA levels (Fig. 3). Quantification of endogenous CO in the murine tissues by the method developed by ourselves^34,35^ showed that endogenous CO levels were significantly reduced in the liver in the first phase (Fig. 5a), whereas they were unchanged in the brains of the hemoCD1-treated mice (Fig. S3). The relationship between the enhanced clock gene expression and the reduced CO level in the liver is interpreted in terms of the CO-responsive function of NPAS2 as previously reported *in vitro*^13^, *i.e*., the binding of the BMAL1:NPAS2 heterodimer to the E-box sequence is facilitated under low CO concentrations.

In the second phase (16.5–19.5), the amount of endogenous CO in the liver of hemoCD1-treated mice increased much larger than those in the controls (Fig. 5a). We have previously demonstrated that endogenous CO in mice is quickly produced by inducing HO-1, an inducible form of HO, when endogenous CO is removed by hemoCD1, whereas HO-2, a constitutive form of HO, is not affected by hemoCD1^34^. Indeed, we found that HO-1 protein expression was strongly induced in the hemoCD1-treated mice at clock time 17.5, leading to the acceleration of endogenous CO production as shown in Figs 5b and S5. The increase in the amount of endogenous CO might cause down-regulation of the clock genes in the second phase, as shown in Fig. 3. Further, we confirmed the CO-dependent activity changes of NPAS2 and CLOCK using chromatin immunoprecipitation (ChIP) analysis with anti-NPAS2 and anti-CLOCK antibodies. The quantification of the amount of DNA bound to BMAL1:NPAS2 and BMAL1:CLOCK in the murine liver is displayed in Fig. 5c,d as a function of clock time. The changes in the quantities of DNA bound to these proteins were inversely correlated with the endogenous CO levels. It should be noted that the expression levels of *Npas2* and *Clock* mRNA were unaffected by administration of hemoCD1 (Fig. S6). Therefore, the significant increases and decreases in the DNA/protein complex levels observed at clock time 14.8 (the first phase) and 17.5 (the second phase) indicate that DNA binding of the BMAL1:CLOCK(NPAS2) heterodimers is modulated by endogenous CO level *in vivo*, resulting in up- and down-regulations of the clock genes.

### CO depletion-induced inflammation affects clock gene expression

**Figure 6:**
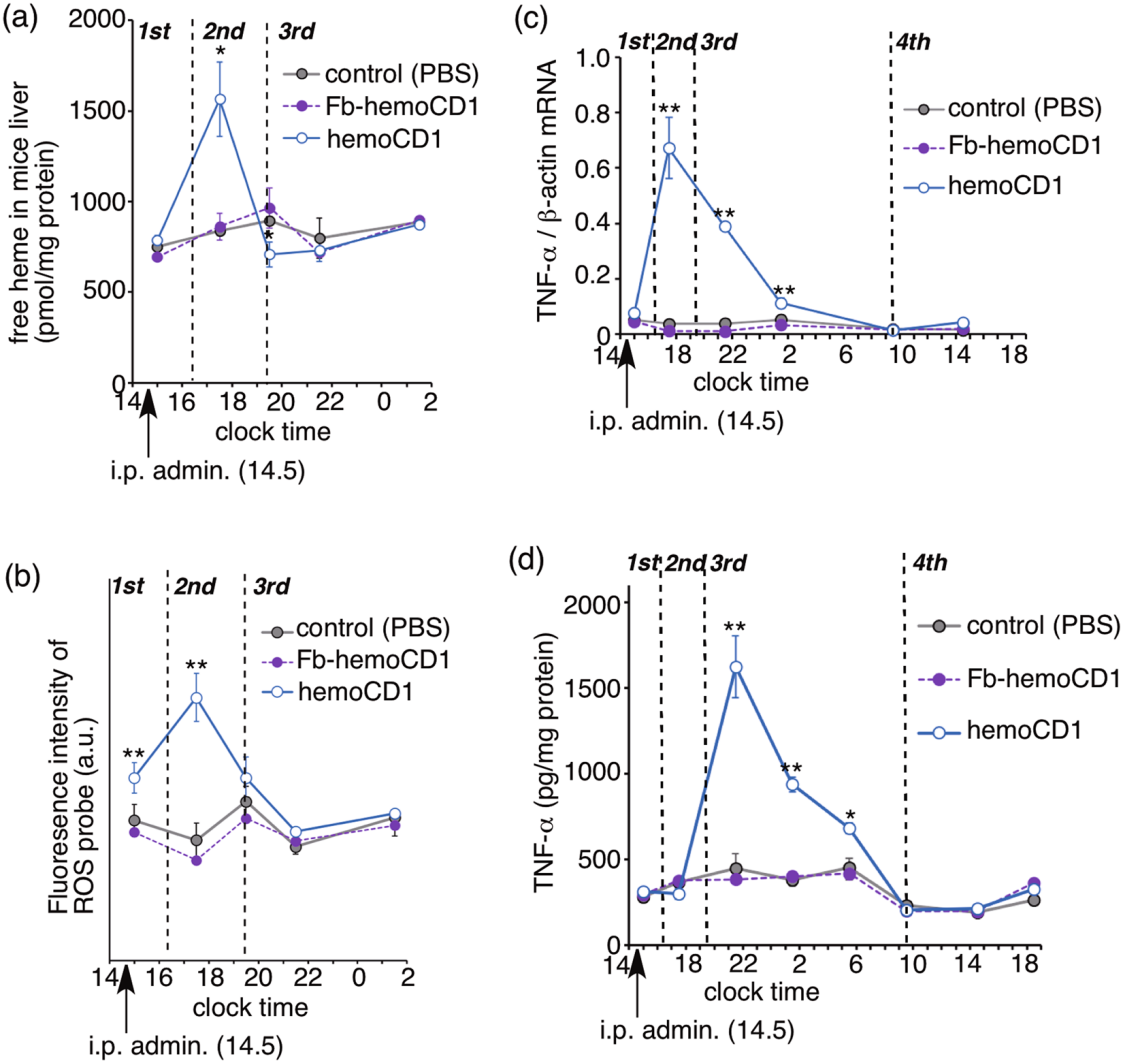
Changes in the amounts of free heme (**a**), ROS (**b**), and TNF-α (mRNA, (**c**); protein, (**d**)) in the liver of hemoCD1-treated mice. PBS and Fb-hemoCD1 were similarly administered as controls. Each bar represents the mean ± SE (*n* = 3 mice per group). Asterisk denotes statistical significance (^*^P < 0.05, ^**^P < 0.01), as compared to the controls.

In the third phase (clock time after 19.5), endogenous CO and DNA/protein complex levels returned to normal (Fig. 5), whereas the levels of the clock genes (*Per1*, *Per2*, *Cry1*, and *Cry2*) were still down-regulated until around 9.5 on day two (Fig. 3). We hypothesized that the down-regulation was ascribed to the inflammatory responses caused by removal of endogenous CO. Our previous study^34^ revealed that the removal of endogenous CO from CO-hemoglobin circulating in blood leads to accumulation of free heme in plasma. Indeed, the amount of free heme was temporally increased in the liver at 17.5 (the second phase) in the liver (Fig. 6a). Simultaneously, reactive oxygen species (ROS) levels in the hemoCD1-treated mice were much higher than those in normal mice (Fig. 6b). Free heme in organs produces ROS *via* a Fenton-type reaction^38^. The ROS-induced oxidative stress enhanced the production of inflammatory cytokines, such as TNF-α (Fig. 6c)^39^. Concurrently, TNF-α significantly accumulated in the hemoCD1-treated mice in the third phase (Fig. 6d). Inflammatory cytokines such as TNF-α affect clock gene expression through their inflammatory cascades^40,41^. Therefore, the long-term down-regulation of the clock genes in the third phase seems to be due to the accumulation of inflammatory cytokines such as TNF-α. TNF-α protein levels returned to normal at clock time 9.5 on day two, at which the clock genes (*Per1*, *Per2*, *Cry1*, and *Cry2*) in the hemoCD1-treated mice recovered to normal (Fig. 3).

**Figure 7:**
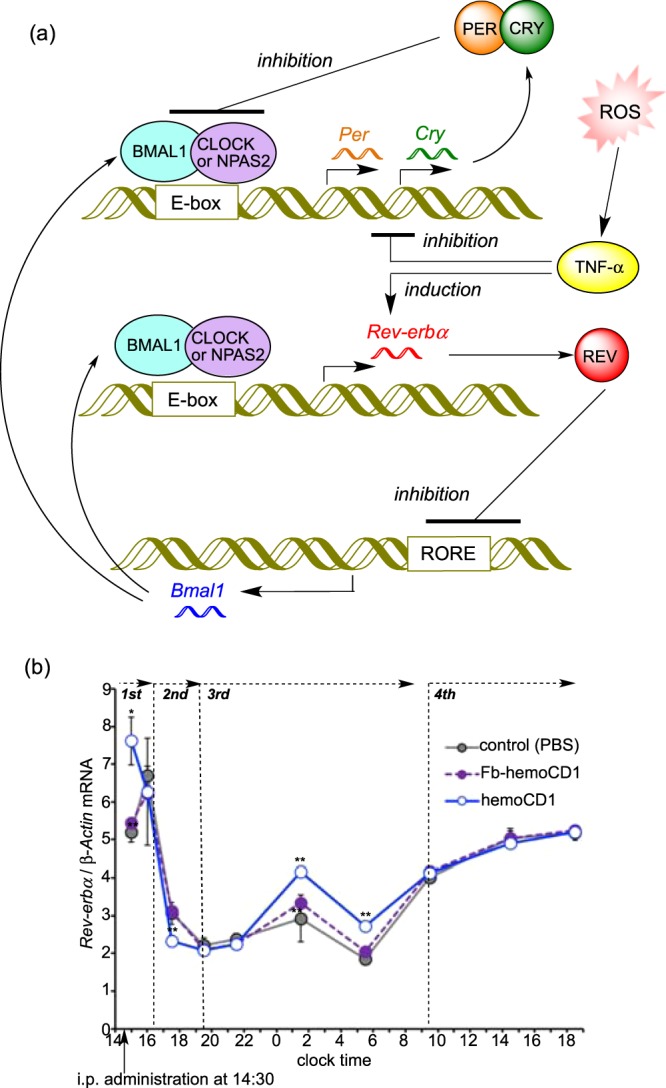
ROS-induced circadian rhythm disruption mediated by TNF-α. (**a**) Schematic representation of the clock system involving *Rev-erbα*. Similar to *Per* and *Cry*, BMAL:CLOCK(NPAS2) enhances transcription of *Rev-erbα*. Additionally, TNF-α, which inhibits *Per* and *Cry*, slightly induces *Rev-erbα*. REV proteins inhibits the expression of BMAL1. (**b**) Changes in the relative mRNA levels of *Rev-erb*α in the murine liver after i.p. administration of hemoCD1. The mice were housed for two weeks under a 12 h light/dark (LD) cycle (lights on at 7:00 and light off at 19:00) until the day before the experiments. The mice were then housed under constant dark (DD) conditions during the observations. PBS and Fb-hemoCD1 were similarly administered as controls. Each bar represents the mean ± SE (*n* = 3 mice per group). Asterisk denotes statistical significance (^*^P < 0.05, ^**^P < 0.01), as compared to the controls. The components of Fig. 7a were drawn using ChemBioDraw.

To confirm the participation of TNF-α in CO removal-induced circadian rhythm disruption, we measured the expression levels of *Rev-erbα*, a circadian component induced by BMAL1:CLOCK(NPAS2)^1–3^. It has been reported that, in contrast to *Per*1/*Per*2, the expression of *Rev-erbα* is slightly enhanced by TNF-α (Fig. 7a)^40,41^. Indeed, the mRNA levels of *Rev-erbα* in the hemoCD1-treated mice were slightly but meaningfully higher than those in the controls at the third phase, whereas up- and down-regulations in the first and second phases were similar to those observed for *Per* and *Cry* (Fig. 7b). This result support a mechanism whereby changes in the mRNA levels in the first and second phases were caused by CO-dependent DNA-binding activity changes in NPAS2 and CLOCK, whereas the changes in the third phase were caused by an inflammatory response. The enhanced *Rev* expression possibly caused down-regulation of BMAL1 in the third phase (Fig. S6)^1–3^, which might have contributed to down-regulation of *Per* and *Cry* in the third phase.

## Discussion

The participation of CO in the circadian clock has been proposed since the important paper on the CO-responsive function of NPAS2 in 2002^13^. However, the study on the physiological contribution of endogenous CO to the circadian clock has been few, probably due to the difficulty in the loss-of-function approach for endogenous CO. The combinatorial use of HO-knockout/knockdown systems with exogenously applied gaseous CO or CO-releasing molecules (CO-RMs) has provided insights into the role of endogenous CO in the circadian clock^24^. The E-box-controlled clock genes are significantly up-regulated in the HO-1-knockout cells, and further up-regulated by knockdown of HO-2. The addition of CO or CO-RMs to the cells partly suppresses the up-regulation. Interestingly, exogenous application of CO to wild-type cells hardly affects the clock gene expression. These findings suggest that endogenous CO is necessary and sufficient for playing a crucial role in regulation of the circadian rhythms. However, the depletion of the HO activity seems to bring the side effects other than CO removal because the restoring effect by exogenous CO is quite limited in the HO-depleted system. As mentioned in the Introduction, inhibition of the HO activity might disturb several biological events, such as heme degradation, production of biliverdin and iron, and an intracellular NADPH concentration. In the present study, we used an endogenous CO-scavenging agent, hemoCD1, to see the effects of endogenous CO removal on the circadian clock of mice. This pseudo-knockdown approach for CO provided the clear evidence that endogenous CO contributes to regulation of the rhythmic expression of clock genes *in vivo*. Upon temporal removal of endogenous CO by hemoCD1, the circadian rhythms of the E-box-controlled clock genes were considerably interfered, and the locomotor activity of the mice was accordingly affected.

**Figure 8:**
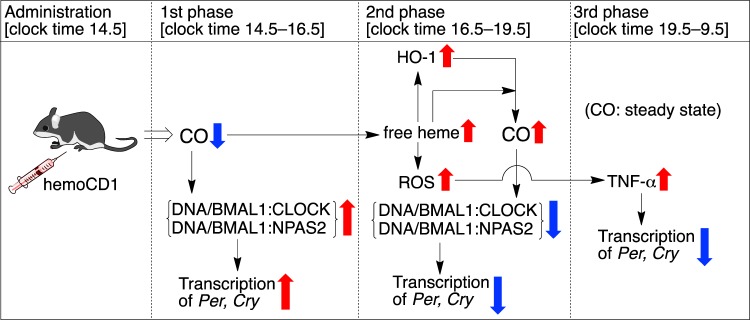
A mechanism for the circadian clock disruption caused by the hemoCD1-mediated endogenous CO depletion in mice. The components of Fig. 8 were drawn using ChemBioDraw.

Figure 8 summarizes a plausible mechanism for the endogenous CO removal-induced circadian clock disruption. The i.p. administration of hemoCD1 immediately lowered endogenous CO levels in the murine liver in the first phase. The removal of endogenous CO facilitated the binding of BMAL1:CLOCK(NPAS2) heterodimers to E-box, resulting in enhancement of transcription of *Per* and *Cry*. This *in vivo* observation is consistent with the CO-responsive DNA-binding function of NPAS2 demonstrated *in vitro*^13^. The heme bound in the heme pocket of the CLOCK PAS-A domain also binds CO *in vitro* similarly to other gas-sensor proteins^19^, although the CO-dependent DNA-binding function has not been confirmed for CLOCK *in vitro*. Unlike NPAS2, it is difficult, as we have also tried several times, to confirm the CO-dependent DNA-binding of CLOCK by means of electrophoretic mobility shift assay, probably due to much lower CO binding affinity of CLOCK (*K*_d_ = *ca*. 0.1 mM at 22 °C)^19^ than that of NPAS2 (*K*_d_ = 1–2 μM at 25 °C)^13^. In contrast, the ChIP experiments for the hemoCD1-treated murine liver (Fig. 5c,d) clearly indicated that the DNA-bindings of BMAL1:CLOCK as well as BMAL1:NPAS2 were significantly altered by internal CO levels. The CO-responsive function of CLOCK has been rarely reported^19,24^, and should be highlighted from the molecular biological point of view, although this would be beyond the scope of this study. We concluded that the up-regulation of the E-box-controlled clock genes observed in the first phase is ascribed to the enhanced transcriptional activities of both BMAL1:NPAS2 and BMAL1:CLOCK at E-box under the CO-reduced conditions.

HemoCD1 administered to mice primarily removes endogenous CO from cell-free CO-hemoglobin in blood, forming cell-free oxy-hemoglobin that is readily oxidized to met-hemoglobin (Fe^III^ state) by ROS in the blood plasma^34^. CO-hemoglobin is resistant against the oxidation mediated by ROS, such as hydrogen peroxide^34^. As cell-free met-hemoglobin easily dissociates to heme and apo-protein^38^, free heme tends to be accumulated in the plasma and tissues in the endogenous CO-removed mice^34^. The accumulated free heme is taken up by hepatic cells^38^ and induces HO-1 *via* activation of Bach-1^9^, resulting in an increase of endogenous CO production *via* heme degradation. Indeed, the amount of endogenous CO is significantly increased in the hemoCD1-treated mice in the second phase (Fig. 6a). The excessively produced endogenous CO might cause additional dissociation of BMAL1:CLOCK(NPAS2) from E-box, resulting in down-regulation of the E-box-regulated clock genes in the second phase. Therefore, the data observed in the first and second phases strongly supports the scenario that the E-box-controlled clock gene transcriptions by both BMAL1:NPAS2 and BMAL1:CLOCK are modulated by internal CO *in vivo*. The expression of HO-1 shows a circadian rhythm^24^. Endogenous CO production must also be circadian, as we show the time-dependent changes in the amounts of endogenous CO in the control groups (Fig. 5a). The circadian production of endogenous CO is possibly related to regulation of the core TTFL through acting on NPAS2 and CLOCK.

Circadian rhythms tend to be disrupted in injured cells and tissues^22,23,41,42^. Increases in inflammatory cytokines, such as IL-1β and TNF-α, affect the clock gene expression and function through acting mainly on the E-box regulatory elements^40,41^ although details have not been fully established. The murine cells treated with TNF-α *in vitro* and *in vivo* cause down-regulation of *Per1*, *Per2*, and other several clock genes, except for *Rev-Erbα*^40^. Interestingly, it has been recently demonstrated that inflammation-induced circadian rhythm disruption is suppressed by application of exogenous CO^22,23^. In contrast, the endogenous CO-reduced mice by hemoCD1 showed an inflammatory response due to accumulation of free heme and ROS, as evidenced by the data in Fig. 6. TNF-α significantly accumulated in the third phase (*ca*. 5 h after the administration of hemoCD1), which might have caused further down-regulation of clock gene (*Per*, *Cry*) expression. The decreased wheel running activity observed in the hemoCD1-treated mice could be ascribed not only to down-regulation of *Cry*^37^ but also to accumulation of TNF-α (40). Considering that hemoCD1 cannot reach the brain, the accumulation of TNF-α is likely the main reason for the decreased wheel running. The accumulated TNF-α can be sustained across the blood-brain barrier, which might affect clock gene expression in the suprachiasmatic nucleus^40^. The down-regulation of *Per* and *Cry* ceased at 19 h after the administration of hemoCD1, at which TNF-α levels also returned to the normal state. Therefore, the temporal reduction of endogenous CO by hemoCD1 in mice has a long-term influence on the circadian clock due to the inflammatory response, and the circadian clock disruption continues until excess TNF-α is completely consumed.

The present pseudo-knockdown study for endogenous CO supports the hypothesis reported in the study using an HO-1 knockout system by Kramer *et al*.^24^. In the HO-1 knockout mice, the E-box-controlled clock gene expression was significantly enhanced due to CLOCK activity changes. This is consistent with our observations of the hemoCD1-treated mice during the first phase. Unlike the reported HO-1 knockout system, our system selectively and temporally reduced the amount of endogenous CO in mice. Thus, the internal CO level can be dynamically altered, as shown in Fig. 5. Another advantage of our system is that hemoCD1 can also be used for internal CO quantification. As discussed in the paper by Kramer *et al*.^24^, the amount of endogenous CO was previously only roughly known *in vivo*. By using hemoCD1, we have succeeded in demonstrating the relation between internal CO levels and the core clock system.

In conclusion, based on the pseudo-knockdown strategy for CO, we identified the contribution of endogenous CO to regulation of the circadian clock *in vivo*. Temporal reduction of endogenous CO in mice by hemoCD1 significantly affected the circadian rhythms of the E-box-controlled clock genes. The mechanistic study suggested that the CO-dependent transcriptional activity changes of NPAS2 and CLOCK and the subsequent inflammatory response to produce TNF-α were both responsible for the CO-removal-induced circadian rhythm disruption. In principle, selective depletion of small biomolecules, such as gaseous signaling molecules, by genetic/pharmacological methods should not be possible without any side effects. We believe that, as demonstrated in this work, the pseudo-knockdown approach based on the highly selective molecular recognition by synthetic compounds will help to clarify the roles and functions of such small molecules in biological systems.

## Materials and Methods

### Preparation of hemoCD1 for administration

HemoCD1 and its free base complex (Fb-hemoCD1) were synthesized in our laboratory^29,34^. The aqueous buffer solution containing the O_2_ adduct of hemoCD1 (1.0 mM in PBS) was prepared according to the method as previously described^34,35^. The solution was used for intraperitoneal (i.p.) administration to mice. The solution of Fb-hemoCD1 was similarly prepared with iron-free porphyrin.

### Animal experiments

All animal experiments were approved by the Animal Experimental Committee of Doshisha University (Approval No. A 16001, A17033, A18040) and carried out in accordance with the Guidelines for Animal Experiments of Doshisha University. Male C57BL/6N mice weighing 20–22 g were used. For acclimatization, the mice were housed under a 12 h/12 h light/dark cycle with free feedings for two weeks before the day in the experiments. The mice were then housed under the dark condition without feeding from the experimental day in order to avoid the entrainment by external stimuli (light and food).

A solution of hemoCD1 (0.15 mL) was intraperitoneally administrated to the subject mouse. The mice were sacrificed by cervical dislocation at different time points. The liver and/or whole brain samples were excised by disposable biopsy punch (5 mm), washed with saline, and soaked in RNA later stabilization reagent (QIAGEN). These were stored at 4 °C until use.

### RT-PCR

Total RNA was isolated from the tissues using an RNA lipid tissue mini kit (QIAGEN) according to the manufacture’s instructions. The experimental procedures were the same as previously described^34,35^. The amounts of total RNA used for reverse-transcription were appropriately adjusted in the range of 2–100 ng to see clear amplifications at the reasonable range in real-time PCR analyses (for *Per1*, *Per2*, *Cry2*, *Clock*, and *Rev-erbα*, 2 ng; for *Cry1* and *Bmal1*, 10 ng; for *Npas2*, 100 ng; for *β-Actin*, 0.32 ng). The primers used in this study are listed in Table S1. Real-time PCR was performed using SYBR Green Master Mix (Applied Biosystems) and a StepOne real-time PCR system. β-Actin was used as a reference gene. All samples were measured in triplicate. Data were analyzed by the relative standard curve method using StepOne software v2.3 (Applied Biosystems).

### Wheel running activity

Wheel running activity of mice was monitored using a RWC-15 automatic system (MELQUEST). For acclimatization, mice were housed in the system with a 12 h/12 h light/dark cycle for one week before being released into constant darkness. The monitoring started at 14:30 and the mice were i.p. administered at 14:30 of the second day. After administration, the monitor was continued for two consecutive days.

### Quantification of endogenous CO

The liver or brain tissues (*ca*. 70 mg per sample, well-flashed to remove blood) were suspended in PBS and then homogenized on ice. The tissue suspensions in PBS (1 mL) were usually separated into 0.9 mL and 0.1 mL for CO and protein quantifications, respectively. The amount of endogenous CO in the tissues was measured by the hemoCD assay^34,35^, where CO was detected by a simple photometric method using hemoCD1. To the tissue suspension (0.9 mL) was added hemoCD1 (1.0 × 10^−8^ mol) in PBS and then sonicated repeatedly on ice. The resulting suspension was filtered using an Amicon Ultra Centrifugal filter unit (MWCO 3000). The filtrate was measured by UV-vis spectrometer (SHIMADZU UV-2450). The calculation for determining the CO content from the absorbances was described previously^34,35^. The CO contents in the tissues were normalized by protein contents determined by a bicinchoninic acid (BCA) method.

### Western Blotting

Lysate of the mice liver was subjected to sodium dodecyl sulfate (SDS)-polyacrylamide gel electrophoresis (PAGE), and the proteins in the gels were electrophoretically transferred onto polyvinylidene difluoride membranes. The experimental procedure was the same as that previously described^34,35^.

### Chromatin Immunoprecipitation (ChIP) assay

The liver tissue (*ca*. 350 mg) was soaked in PBS containing 1% formaldehyde (10 mL/g) and shaken for 10 min at room temperature. To the solution was added glycine to a final concentration of 0.125 M and then incubated for additional 5 min. After homogenization of the tissue, the cells were collected by centrifugation, counted, and then suspended in Lysis buffer (ATTO, 0.75 mL/10^7^ cells) containing 1% protease inhibitor and 0.5 mM phenylmehtylsulfonyl fluoride (PMSF). The cells were then disrupted by sonication (QSONICA, amplitude; 20%, ten times for 20 s each with 40 s intervals). The aliquot sample (20–40 μL) was used for an BCA protein quantification assay. Another aliquot (100 μL) was used for checking the chromatin size as follows; the aliquot solution was mixed with 5 M NaCl aqueous solution (5 μL), heated at 65 °C for 5 h to reverse the cross-linking, and then the solution was heated at 45 °C for 1 h after the additions of RNase A (Wako, 10 mg/mL, 2 μL) and proteinase K (Wako, 20 mg/mL, 2 μL) to remove RNA and proteins. The DNA component in the solution was isolated using QIAquick PCR purification kit (QIAGEN), and then the chromatin size was checked by agarose electrophoresis to ensure that the average size was between 100 and 1000 bp. The sonication was repeated until this size distribution was achieved.

The sonicated chromatin sample was diluted with Lysis buffer (ATTO) at the concentration of 1.25 mg protein/mL and then mixed with protein A/G plus agarose (Santa Cruz Biotechnology) bead slurry (30 μL). After shaking the solution at 4 °C for 2 h, the solution was centrifuged, and the supernatant was collected. An aliquot (20 μL) of the supernatant was separated for the use as the input sample, and therefore stored at −20 °C until the reverse cross-linking. The residual supernatant solution (410 μL) was transferred to a new tube, and mixed with anti-NPAS2 antibody (3 μL, H-270, Santa Cruz Biotechnology) or anti-CLOCK-antibody (5 μL, ab3517, abcam). After the solution was gently shaken overnight at 4 °C, the solution was then mixed with protein A/G plus agarose bead slurry (30 μL) that was preliminarily blocked with salmon sperm DNA. After further incubation for 2 h at 4 °C, the beads were collected by centrifugation, and washed repeatedly with low salt buffer (20 mM Tris-HCl, pH 8, 2 mM EDTA, 1%Triton X-100, 0.1% SDS, 200 μL), high salt buffer (low salt buffer plus 0.5 M NaCl, 200 μL), and LiCl salt buffers (0.25 M LiCl, 10 mM Tris-HCl, pH 8, 1 mM EDTA, 1% sodium deoxycholate, 1% NP-40, 200 μL). The bound materials on the beads were eluted by heating at 65 °C for 20 min with an Elution buffer (70 mM Tris-HCl, pH 8, 1 mM EDTA, 1.5% SDS, 120 μL). After centrifugation, the supernatant (500 μL) was collected, mixed with 5 M NaCl aqueous solution (5 μL), and then heated at 65 °C for 5 h to reverse the cross-linking. After that, the solution was heated at 45 °C for 1 h with RNase A (10 mg/mL, 2 μL) and proteinase K (20 mg/mL, 2 μL) to remove RNA and proteins. The DNA component in the resulting solution was isolated using QIAquick PCR purification kit (QIAGEN).

The resulting DNA samples were quantified by real-time PCR. The primers used in the ChIP assay are listed in Table S2. Real-time PCR was performed using SYBR Green Master Mix (Applied Biosystems) and a Step One real-time PCR system. β-Actin was used as a reference gene. All samples were measured in triplicate. Data were analyzed by the relative standard curve method using StepOne software v2.3 (Applied Biosystems).

### Quantification of free heme

The liver tissues (*ca*. 200 mg) was homogenized with RIPA lysis buffer (500 μL, ATTO, 20 mM HEPES, 150 mM NaCl, 1.0% IGEPAL CA- 630, 0.1% SDS, and 0.5% sodium deoxycholate). The supernatant was obtained by centrifugation. Aliquot was used for BCA protein quantification. The residual supernatant was passed through an Amicon Ultra Centrifugal filter units (MWCO 3000) to remove proteins. The concentration of free hemin in the protein-depleted solution was quantified by a chromogenic assay according to the manufacturer’s instructions (QuantiChrom heme assay kit, Bioassay Systems). The colorimetric change was monitored at 405 nm using a Filter Max F5 plate reader (Molecular Devices).

### Quantification of ROS

The plasma samples were collected from the hemoCD1-treated mice blood after centrifugation (4000 *g*, 20 min). The tissue samples were also collected from the mice liver (*ca*. 50 mg). The tissue was mixed with buffer (1 mL) containing 150 mM KCl, 20 mM Tris, 0.5 mM EDTA, 5 mM glucose, 1 mM MgCl_2_, 0.5 mM octanoic acid. After homogenization, the tissue samples were diluted to the 50 mg protein/mL concentrations, and then mixed with the solution of dihydrorhodamine (2 μL, 10 mM DMSO solution) as a fluorescent ROS probe. After removing insoluble materials by centrifugation, fluorescence intensity of the supernatant (100 μL each) was detected using a Filter Max F5 plate reader (Molecular Devices, λ_ex_ = 485 ± 20 nm and λ_em_ = 535 ± 25 nm).

### Quantification of TNF-α protein

The mouse liver was homogenized in RIPA Lysis Buffer (ATTO, 20 mM HEPES, 150 mM NaCl, 1.0% IGEPAL CA- 630, 0.1% SDS, and 0.5% sodium deoxycholate) after the additions of the protease inhibitor (ATTO) and phosphatase inhibitor (ATTO). The total protein concentration was determined by an BCA assay. The amount of TNF-α protein in the liver was determined using a mouse TNF-α ELISA kit (Novex Life Technologies). The experiments were performed according to the manufacture’s instruction. The amount of TNF-α was quantified by a photometric measurement using a Filter Max F5 plate reader (Molecular Devices, Abs. at 450 nm).

### Statistical analysis

All data represent the means ± SE from at least three different experiments. Statistical analysis (*n* ≥ 3) was performed by using an unpaired Student’s *t*-test. The *P* value less than 0.05 was considered significant.

## Electronic supplementary material

Supplementary Information
