# Supplementary material for: Circadian clock disruption by selective removal of endogenous carbon monoxide
Source: Sci Rep. 2018 Aug 10;8:11996. doi: 10.1038/s41598-018-30425-6 (PMC6086871; doi:10.1038/s41598-018-30425-6)
Supplement: Supplementary file 1 — Supplementary Information [file 41598_2018_30425_MOESM1_ESM.pdf]

Supplementary Information for

Circadian clock disruption by selective removal of endogenous carbon monoxide

Saika Minegishi, Ikuko Sagami, Shigeru Negi, Koji Kano, and Hiroaki Kitagishi[1]

[1]To whom correspondence should be addressed. E-mail: hkitagis@mail.doshisha.ac.jp

Table S1. List of primers used for real-time PCR.

gene	primers	length (bp)
Per1	for 5'- AGAAGAAAACAGCACCAGCT -3'	99
	rev 5'- TCTTGAGTTATAAGAACCCCA -3'	
Per2	for 5'- ATGCTCGCCATCCACAAGA -3'	72
	rev 5'- GCGGAATCGAATGGGAGAAT -3'	
Cry1	for 5'- CTGGCGTGGAAGTCATCGT -3'	77
	rev 5'- CTGTCCGCCATTGAGTTCTAT -3'	
Cry2	for 5'- AAGCTGAATTCGCGTCTGTT -3'	238
	rev 5'- GTGGTTTCTGCCCATTCAGT -3'	
Npas2	for 5'- ACGCAGATGTTCGAGTGGAAA -3'	138
	rev 5'- CGCCCATGTCAAGTGCATT -3'	
Clock	for 5'- CGGCGAGAACTTGGCATT -3'	135
	rev 5'- AGGAGTTGGGCTGTGATCA -3'	
Bmal1	for 5'- GCAGTGCCACTGACTACCAAGA -3'	201
	rev 5'- TCCTGGACATTGCATTGCAT -3'	
Rev-erbα	for 5'- CGTTCGCATCAATCGCAACC -3'	203
	rev 5'- GATGTGGAGTAGGTGAGGTC -3'	
β-actin	for 5'- AGAGGGAAATCGTGCGTGAC -3'	138
	rev 5'- CAATAGTGATGACCTGGCCGT -3'	

Table S2. List of primers used for real-time PCR in the ChIP assay.

ChIP system	*gene*	primers
anti-NPAS2 antibody		*Per2* for 5'- AGAAAAGCCCTGCTGTTCCA -3'
		rev 5'- CCAAATGCGGTGGTGTAGTTT -3'
anti-CLOCK antibody		*Per1* for 5'- GGGTAGTTTCCCTCCCTCAC -3'
		rev 5'- TGGCATCTGATTGGCTACTG -3'

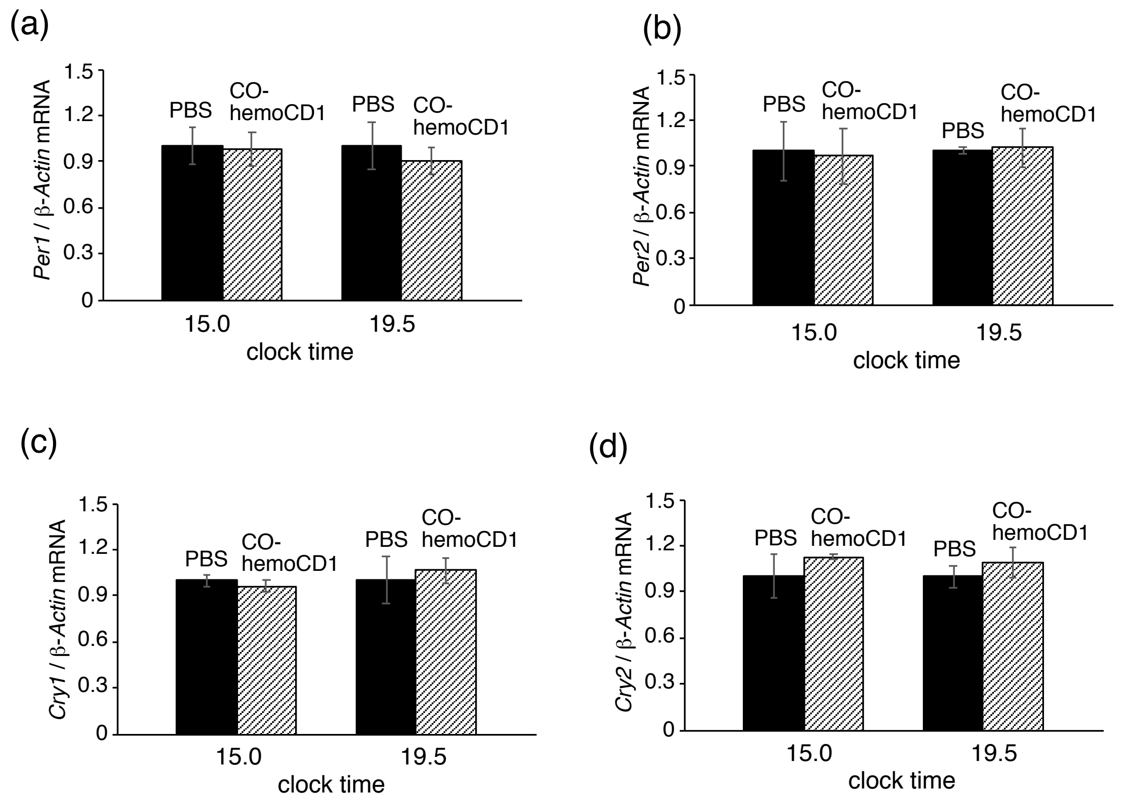

Figure S1. The relative mRNA levels of *Per1* (a), *Per2* (b), *Cry1* (c), and *Cry2* (d) at clock time 15.0 and 19.5 in the liver of mice after the intraperitoneal administration of CO-hemoCD1 at clock time 14.5. Before one day from the experiments, the mice were housed for two weeks under the light/dark (LD) cycles (light on at 07:00 and light off at 19:00). The mice were then housed under the constant dark (DD) conditions during the observations. PBS was similarly administered as controls. Each bar represents the means ± SE (n = 3 mice per group). There are no significant changes by CO-hemoCD1, indicating that the clock gene expression changes observed in hemoCD1-treated mice (Figure 3) are caused by selective CO-depletion in mice.

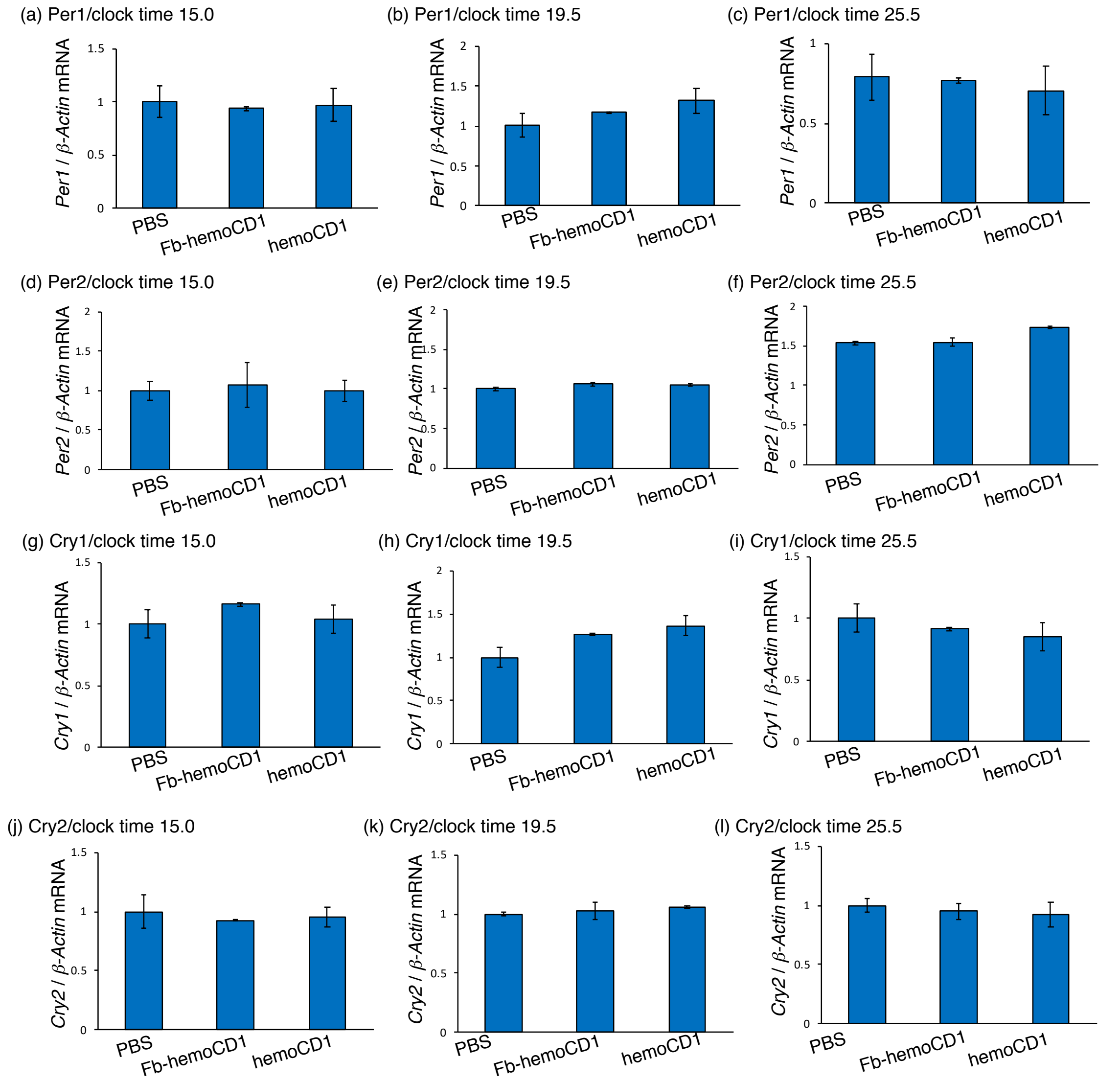

Figure S2. The relative mRNA levels of *Per1* (a–c), *Per2* (d–f), *Cry1* (g–i), and *Cry2* (j–l) at clock time 15.0, 19.5 and 25.5 (1.5 on day two) in the whole brain of mice after the intraperitoneal administration of hemoCD1 at CT 7.5. Before one day from the experiments, the mice were housed for two weeks under the light/dark (LD) cycles (light on at 07:00 and light off at 19:00). The mice were then housed under the constant dark (DD) conditions during the observations. PBS and Fb-hemoCD1 were similarly administered as controls. Each bar represents the means ± SE (n = 3 mice per group). There are no significant changes between hemoCD1 and controls, indicating that the i.p. injection of hemoCD1 to mice does not affect the circadian system in brain probably due to low permeability of hemoCD1 to blood brain barrier.

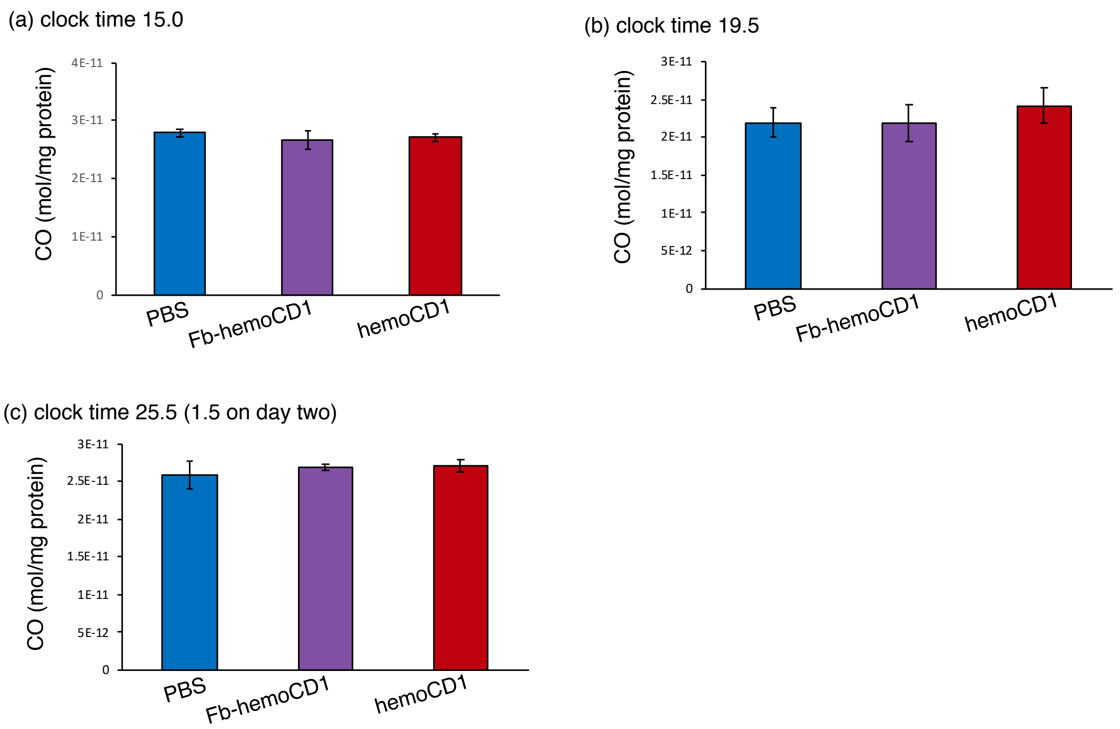

Figure S3. The amount of endogenous CO at clock time 15.0 (a), 19.5 (b), and 25.5 (c) in the whole brain of the hemoCD1-administerd mice at clock time 14.5. The amount of CO was quantified by the assay using hemoCD1 (see Experimental section). Each bar represents the means ± SE (n = 3 mice per group). Similar to the mRNA levels of clock genes shown in Figure S2, there are no significant changes between hemoCD1 and controls due to low permeability of hemoCD1 to blood brain barrier.

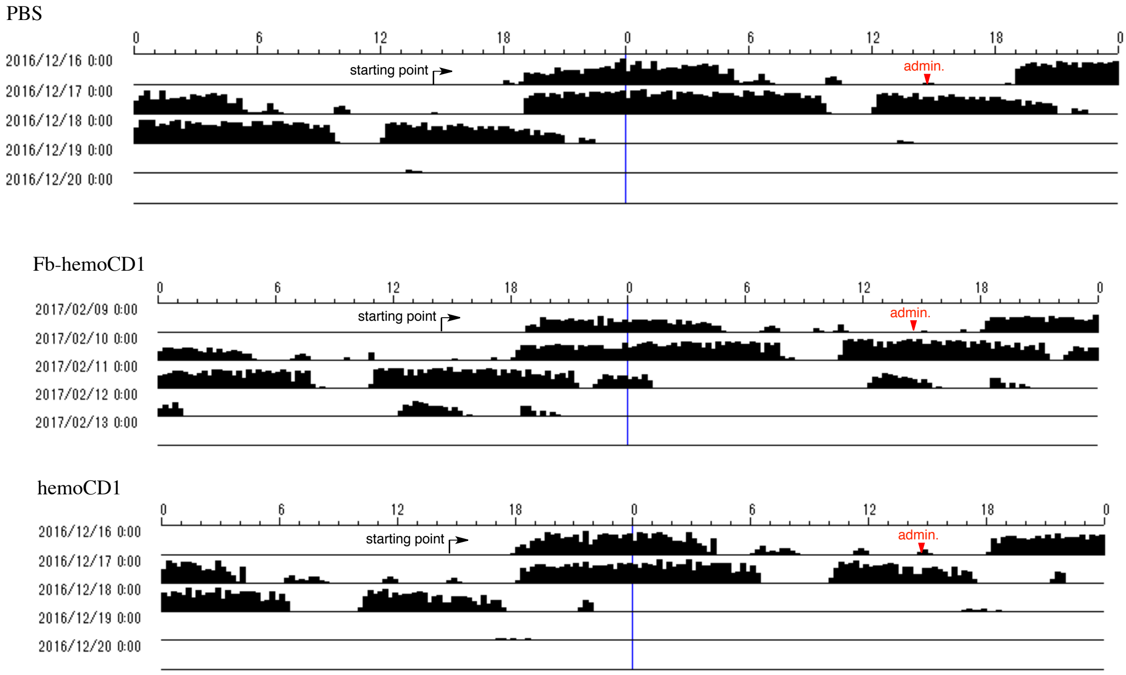

Figure S4. The double-plotted actograms for the wheel-running activity of the mice under the constant dark conditions before and after the i.p. administration of PBS, Fb-hemoCD1, and hemoCD1 at clock time 14.5. Note that the activity on day three of all cases decreased due to restricted feeding to the mice.

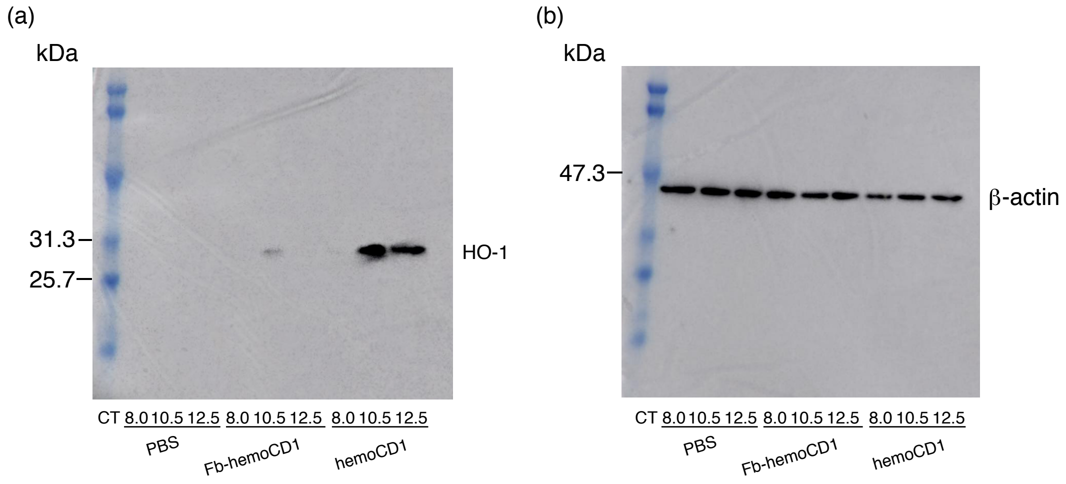

Figure S5. Full length western blots for HO-1and β-actin for Fig. 5b.

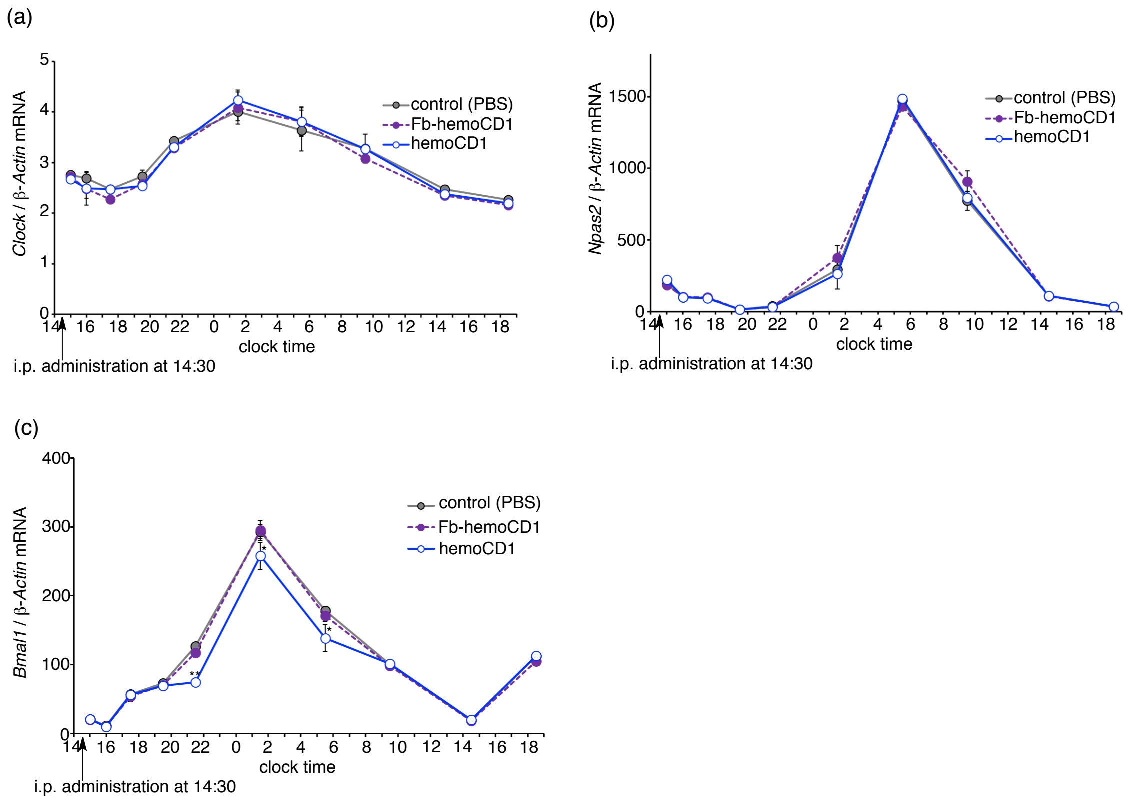

Figure S6. Changes in the relative mRNA levels of *Clock* (a), *Npas2* (b), and *Bmal1* (c) in the murine liver after the intraperitoneal administration of hemoCD1. Before one day from the experiments, the mice were housed for two weeks under the light/dark (LD) cycles (light on at 07:00 and light off at 19:00). The mice were then housed under the constant dark (DD) conditions during the observations. PBS and Fb-hemoCD1 were similarly administered as controls. Each bar represents the means ± SE (n = 3 mice per group). Asterisk denotes statistical significance (*$P < 0.05$, **$P < 0.01$), as compared to the controls. Note that the value on the vertical axis cannot be directly compared between the different panels (a–c) because the amount of cDNA used for real-time PCR varies with the gene of interest, see Experimental section. These results indicate that the mRNA expression levels of CLOCK and NPAS2 are not affected by the removal of endogenous CO, while these transcriptional activities are changed as measured by ChIP assay (see Figure 5c, d). The mRNA level of BMAL1 was also not significantly affected by the CO-removal, although slight down-regulation was observed during 19.5–9.5 due to the slight up-regulation of *Rev-erbα* by TNF-α (see Figure 7).
